# A common variant of endothelial nitric oxide synthase (Glu298Asp) is associated with collateral development in patients with chronic coronary occlusions

**DOI:** 10.1186/1471-2261-5-27

**Published:** 2005-09-15

**Authors:** Nicolas Lamblin, François J Cuilleret, Nicole Helbecque, Jean Dallongeville, Jean-Marc Lablanche, Philippe Amouyel, Christophe Bauters, Eric Van Belle

**Affiliations:** 1 Hôpital Cardiologique, Centre Hospitalier Universitaire de Lille, Place de Verdun, 59037 Lille cedex, France; 2 INSERM U508, Institut Pasteur de Lille, 1 rue Calmette, 59019 Lille cedex, France

## Abstract

**Background:**

Experimental studies support an important role for endothelial nitric oxide synthase (eNOS) in the regulation of angiogenesis. In humans, a common polymorphism exists in the eNOS gene that results in the conversion of glutamate to aspartate for codon 298. *In vitro *and *in vivo *studies have suggested a decreased NOS activity in patients with the Asp^298 ^variant. We hypothesized that a genetic-mediated decreased eNOS activity may limit collateral development in patients with chronic coronary occlusions.

**Methods:**

We selected 291 consecutive patients who underwent coronary angiography and who had at least one chronic (>15 days) total coronary occlusion. Collateral development was graded angiographically using two different methods: the collateral flow grade and the recipient filling grade. Genomic DNA was extracted from white blood cells and genotyping was performed using previously published techniques.

**Results:**

Collateral development was lower in patients carrying the Asp^298 ^variant than in Glu-Glu homozygotes (collateral flow grade: 2.64 ± 0.08 and 2.89 ± 0.08, respectively, p = 0.04; recipient filling grade: 3.00 ± 0.08 and 3.24 ± 0.07, respectively, p = 0.04). By multivariable analysis, three variables were independently associated with the collateral flow grade: female gender, smoking, and the Asp^298 ^variant (p = 0.03) while the Asp^298 ^variant was the sole variable independently associated with the recipient filling grade (p = 0.03).

**Conclusion:**

Collateral development is lower in patients with the Asp^298 ^variant. This may be explained by the decreased NOS activity in patients with the Asp^298 ^variant. Further studies will have to determine whether increasing eNOS activity in humans is associated with coronary collateral development.

## Background

In spite of recent advances in the techniques used for myocardial revascularization, chronic total coronary occlusions are frequently observed in patients with coronary artery disease. This could lead to symptoms of angina, quality of life impairment, left ventricular dysfunction, and prognosis worsening. In the case of severe stenosis or total occlusion of a coronary artery, the collateral circulation may be an alternative source of blood supply to the myocardium at risk [1,2]. Although some factors, such as the duration of myocardial ischemic symptoms, have been associated with the extent of collateralization, coronary collateral development remains difficult to anticipate and there is considerable inter-individual variability in this process [3]. One emerging concept in cardiovascular diseases, which could explain this variability, is the possible interaction between genetic determinants and the pathophysiological responses to cardiac injury.

Among candidate genes that may be implicated in collateral development is the endothelial nitric oxide synthase (eNOS) gene. Experimental studies support an important role for eNOS in the regulation of angiogenesis [4]: mice lacking eNOS gene have severely reduced angiogenesis in response to tissue ischemia [5,6] while eNOS overexpression enhances angiogenesis [7-9]. In humans, different common polymorphisms exist in the eNOS gene and among them one that results in the conversion of glutamate to aspartate for codon 298. *In vitro *studies have suggested that the Asp^298 ^variant may be functional and associated with a decreased of eNOS activity [10]. *In vivo *studies have documented an increased reactivity to alpha-adrenergic stimulation in patients with the Asp^298 ^variant suggesting a decreased NOS activity [11].

In the present study, we hypothesized that a genetic-mediated decreased eNOS activity may limit collateral development in patients with chronic coronary occlusions. We studied 291 patients with chronic coronary occlusions in whom collateral development was graded angiographically. We show that patients with the Asp^298 ^variant have significantly less collateral vessel formation than Glu-Glu homozygotes.

## Methods

### Study population

Between May 2000 and October 2001, 2050 consecutive patients who underwent a coronary angiography at our institution were enrolled in a registry. All patients gave informed consent and had blood and serum samples that were stored at -80°C until further analysis. The baseline clinical and angiographic characteristics were prospectively recorded by trained physicians.

For the purpose of this study, we selected all patients who had at least one chronic (>15 days) total occlusion of a major coronary vessel. The patients with a history of coronary artery bypass graft were excluded. Two hundred and ninety one patients were thus selected to form the study population.

### Angiography procedure and coronary collaterals grading

Selective coronary angiography was performed in multiple orthogonal projections using the Judkins technique. In case of significant lesion (stenosis or total occlusion), there was an intracoronary nitrates infusion. Collateral development was graded using two different methods by two independent observers. These methods have been previously validated [12].

The *collateral flow grade *evaluates the flow in the collateral: *0 *= no flow in the collateral; *1 *= the collateral is barely apparent; dye is not visible throughout the cardiac cycle but is present in at least 3 consecutive frames; *2 *= the collateral is moderately opaque but is present throughout at 75% of the cardiac cycle; *3 *= the collateral is well opacified and the column of dye is well defined but is < 0.7 mm wide throughout the majority of its length; *4 *= the collateral is well opacified, fills antegrade, and is very large.

The second method was the *recipient filling grade*: *0 *= no angiographically apparent collaterals; *1 *= apparent collaterals extend into a region of myocardium with no angiographically apparent recipient vessel; *2 *= minimal recipient filling by collaterals is manifested by minor side branch filling and no epicardial artery or epicardial side branch filling; *3 *= Moderate recipient filling by collaterals is manifested by complete filling of epicardial side branches and partial filling of a major epicardial artery; *4 *= there is complete filling of a major epicardial segment.

### Genetic analysis

Blood samples were collected at the time of coronary angiography. Genomic DNA was extracted from white blood cells by a « salting out » procedure as previously described [13]. DNA fragment – including the G/T translation in the exon 7 – amplification was performed by Polymerase Chain Reaction (PCR). Primers and PCR conditions used for eNOS have been reported previously [14]. The products was digested by the Ban II enzyme for genotyping as previously described [14] and the results of the genotyping were tested for the Hardy-Weinberg equilibrium (p > 0.05).

### Statistical analysis

Patients were grouped on the basis of the presence or absence of the Asp^298 ^variant (Asp^298 ^homozygotes and heterozygotes were combined and compared to the homozygotes Glu^298^) as previously reported [11,14-16]. For continuous variable, distributions were first tested before analyses. Since the distribution were normal, they were presented as mean ± SEM and were compared with use of the bilateral unpaired Student' t test. Qualitative variables were compared with use of the Pearson chi-square test or the Fisher exact test when necessary. Multivariable analysis was performed with use of a general linear model (GLM) adjusted for age, gender, smoking, hypertension, hypercholesterolemia and diabetes mellitus. Statistical analysis were performed with the SAS software, version 8 (SAS Institute Inc., Cary, NC, USA).

## Results

The baseline characteristics of the study population are shown in table 1. There were no statistically significant differences between patients with the Asp^298 ^variant and Glu-Glu homozygotes. Most patients were male with a mean age of 63 ± 11 years and with a high prevalence of cardiovascular risk factors. Notably, 77% of the patients were current or past smokers and 36% were diabetics. Cardiovascular medications did not differ between the two groups. The angiographic severity of coronary atherosclerosis was similar in the two groups. In most of the cases, only one coronary artery was totally occluded.

**Table 1:** Demographics and medical therapy at baseline by genotype

	Asp^298 ^(n = 168)	Glu-Glu (n = 123)	All patients (n = 291)	*p*
Age, years	63 ± 11	62 ± 11	63 ± 11	0.92
				
Female gender, %	18	20	19	0.72
				
Body mass index, kg/m^2^	28.3 ± 4.7	27.8 ± 4.6	28.1 ± 4.6	0.32
				
Risk factors, %				
Smoking	76	78	77	0.71
Hypercholesterolemia	76	81	78	0.29
Hypertension	54	55	55	0.86
Diabetes mellitus	35	37	36	0.80
Familial history of CAD	37	35	36	0.18
				
Clinical symptoms, %				
Stable	73	78	75	0.35
Unstable	27	22	25	
				
Angiographic data:				
No. of vessels with > 50% stenosis, %				
1 vessel	24	25	25	
2 vessels	38	34	36	0.92
3 vessels	38	41	39	
				
No. of vessels with total occlusion, %				
1 vessel	85	83	84	0.61
2 vessels	15	17	16	
				
Occluded LAD, %	38	29	34	0.12
Occluded Cx, %	17	22	19	0.32
Occluded RCA, %	60	66	62	0.27
				
Cardiovascular medications, %				
ASA	78	78	78	0.96
ACE inhibitors	55	52	54	0.57
ARB	6	10	8	0.24
Beta-blockers	63	58	61	0.34
Nitrates	58	48	54	0.09
Calcium antagonists	24	31	27	0.20
Statins	63	58	61	0.34

Data are presented as percent of patients or mean value ± SD

CAD = coronary artery disease; MI, myocardial infarction

LAD = left anterior descending artery; Cx = circumflex; RCA = right coronary artery

ASA = acetylsalicylic acid; ACE = angiotensin-converting enzyme; ARB = angiotensin 2 receptor blockers.

In the overall study population, the mean collateral flow grade was 2.75 ± 0.06 and the mean recipient filling grade was 3.10 ± 0.06. By univariable analysis, angiographic evidence of collateral development was lower in patients carrying the Asp^298 ^variant than in Glu-Glu homozygotes (collateral flow grade: 2.64 ± 0.08 and 2.89 ± 0.08, respectively, p = 0.04; recipient filling grade: 3.00 ± 0.08 and 3.24 ± 0.07, respectively, p = 0.04). When patients were classified into 3 groups (Glu-Glu homozygotes, Glu-Asp heterozygotes, Asp-Asp homozygotes), respective values for collateral flow grade were 2.84 ± 0.08, 2.63 ± 0.09, and 2.69 ± 0.23, while respective values for recipient filling grade were 3.24 ± 0.07, 3.01 ± 0.08, and 2.94 ± 0.23. Independent predictors of collateral development were then determined by multivariable analysis (table 2). When considering the collateral flow grade, three variables were independently associated with an impaired collateral development: female gender, smoking, and the Asp^298 ^variant; there was also a strong trend for a deleterious effect of diabetes mellitus on collateral development. When considering the recipient filling grade, the Asp^298 ^variant was the sole variable independently associated with an impaired collateral development; again, the presence of diabetes mellitus was associated with a trend for a deleterious effect. The presence of the Asp^298 ^variant was therefore the sole independent predictor of both the collateral flow grade and the recipient filling grade.

**Table 2:** Predictors of collateral development: multivariable analysis

	Collateral flow grade		Recipient filling grade	
	β	*p*	β	*p*
Age ≥ 63	- 0.04	0.72	+ 0.03	0.77
Female gender	- 0.49	0.009	- 0.22	0.23
Smoking	- 0.34	0.05	- 0.12	0.47
Hypertension	- 0.04	0.72	- 0.04	0.74
Hypercholesterolemia	- 0.01	1.00	- 0.16	0.23
Diabetes mellitus	- 0.23	0.06	- 0.20	0.09
Asp^298 ^variant	- 0.26	0.03	- 0.24	0.03

Since collateral vessel development has been associated with a preserved left ventricular function in the case of total occlusion of a coronary artery [17], we compared the left ventricular ejection fraction in both groups. A recent evaluation of left ventricular ejection fraction was available in 162 (96%) of patients with the Asp^298 ^variant and in 120 (98%) of Glu-Glu homozygotes. The mean ( ± SD) left ventricular ejection fraction was 53 ± 16% in the overall study population and was 50 ± 16% in patients with the Asp^298 ^variant versus 54 ± 16% in Glu-Glu homozygotes (p = 0.05).

## Discussion

In the present study, we found that patients carrying the Asp^298 ^variant of eNOS gene had significantly less angiographic evidence of collateral vessel formation in response to total coronary occlusion. Multivariable analysis showed that this effect was independent of other factors that influence collateral vessel formation.

Nitric oxide (NO), constitutively produced by endothelial nitric oxide synthase (eNOS), plays critical roles in vascular biology, including regulation of vascular tone and blood pressure. In addition to its vasodilatory properties, NO has been implicated in the modulation of angiogenesis [4]. Ziche et al. suggested that NO may play a role in angiogenesis elicited by VEGF but not by FGF [18]. Murohara et al. demonstrated that angiogenesis developing in response to limb ischemia was severely reduced in mice lacking eNOS gene [5]. Moreover, eNOS over expression in transgenic mice [8] or using gene transfer strategies [7,9] enhances angiogenesis in response to tissue ischemia. There is thus strong experimental evidence to support an important role for eNOS in the regulation of angiogenesis in animal models, however the implication of eNOS activity in collateral vessel formation in response to myocardial ischemia in humans remains unknown

ENOS is encoded by a 26-exon gene located on chromosome 7 [19]. In view of the physiological and pathophysiological importance of NO, the potential role of eNOS in the pathogenesis of various human diseases has been examined using its polymorphic variants as potential disease markers. Different common polymorphisms exist and among them one in nucleotide 894 (G-T) that results in the conversion of glutamate to aspartate for codon 298. A study by Philip et al. has documented *in vivo *an increased reactivity to alpha-adrenergic stimulation in patients with the Asp^298 ^variant suggesting a decreased NOS activity [11]. In recent clinical studies, the Asp^298 ^variant has been implicated as a risk factor for coronary artery disease [20], hypertension [16], or has been associated with a poorer event-free survival in patients with congestive heart failure [14]. In a meta-analysis of 26 studies involving 23028 subjects, homozygosity for Asp^298 ^was associated with increased risk of ischemic heart disease by 31% [15]. When taken together with the results of the present study, the above described literature suggests that a decreased NOS activity in coronary vessels of patients with the Asp^298 ^variant may explain the decreased collateral vessel formation observed in this subgroup of patients. The mechanism by which the Asp^298 ^variant could decrease the eNOS activity remains unclear. The Asp^298 ^variant has been associated with an increased susceptibility to enzymatic cleavage [10] but it has been suggested that the increased susceptibility to proteolytic cleavage of NOS could result from sample preparation [21]. In addition, other recent studies failed to find any association between the Asp298 variant and eNOS activity [22,23]. An other possible explanation is that the variant Asp298 may simply be a genetic marker. For example, the Asp298 variant is in linkage disequilibrium with an other polymorphism resulting in a nucleotide substitution in the promoter region (T/C -786) of the eNOS gene [24,25]. It has been shown that the rarer variant (C) suppresses eNOS transcription by approximately 50%. The Asp298 could be a marker of the occurrence of the unfavorable "C" allele in the promoter region, the latter responsible for the decreased of eNOS activity.

In the present study, collateral vessel formation was assessed using angiographic criteria. Angiographically visible collaterals represent only a fraction of the total collateral vessels because collaterals are angiographically demonstrable only when they reach 200 μm [12]. Several studies have shown that assessing the collateral circulation by intracoronary Doppler flow or pressure wires may be an interesting alternative to determine collateral blood flow in humans [26,27]; however, the invasive nature of this method which imply to cross the occlusion site by a guide wire would limit its interest in a genetic association study like the present one in which inclusion of consecutive and as much as possible unselected cases is mandatory to provide unbiased results. In an attempt to provide a rigorous, systematic analysis of human coronary angiogenesis by angiography, we used two criteria recently reviewed by Gibson et al. [12]. The collateral flow grade focuses on the development of the collateral network itself while the recipient filling grade is adapted from the Rentrop grade [28] and provides information on how the recipient vessel is filled by the collaterals. In the present study, the fact that the deleterious impact of the Asp^298 ^variant was evident with both criteria reinforces our findings. An important question in the present study is to know whether the better angiographic criteria in Glu-Glu homozygotes are a result of the vasodilation properties of eNOS or are due to an increase in blood vessel growth. Such a question is beyond the scope of this clinical study but a direct effect of eNOS on angiogenesis has been documented in the above described experimental studies [7-9]. Moreover, the trend for higher left ventricular ejection fraction observed in Glu-Glu homozygotes suggests a beneficial effect of the collaterals on myocardial function. Finally, our analysis was based on a single time point; further assessment of collateral development by repeated angiographic follow-up would be of interest but was not performed due to the invasive nature of coronary angiography.

## Conclusion

In conclusion, this investigation is the first study to show the relationship between the 894 (G-T) eNOS polymorphism and coronary collaterals in humans. It demonstrates that collateral development is poorer in patients with the Asp^298 ^variant. This may be explained by a decreased NOS activity in patients with the Asp^298 ^variant. Further studies will have to determine whether increasing eNOS activity in humans is associated with coronary collateral development.

## Abbreviations

eNOS: endothelial nitric oxyde synthase

FGF: fibroblast growth factor

PCR: polymerase chain reaction

VEGF: vascular endothelial growth factor

## Competing interests

The author(s) declare that they have no competing interests.

## Authors' contributions

NL, FJC, and EVB participated in the clinical organisation including patients inclusion and angiographic analyses of the collateral development. NL, and NH carried out the genetic analyses and the validation of the analyses. NL performed the interpretation and the statistical analyses of the data. JML, JD, and EVB participated in the conception and the design of the study. PA participated in the interpretation and the analyses of the data. NL, CB, and EVB participated in the conception and design of the study and drafted the manuscript. All authors read and approved the final manuscript.

## Pre-publication history

The pre-publication history for this paper can be accessed here:



## References

[B1] Helfant RH, Vokonas PS, Gorlin R (1971). Functional importance of the human coronary collateral circulation. N Engl J Med.

[B2] Fuster V, Frye RL, Kennedy MA, Connolly DC, Mankin HT (1979). The role of collateral circulation in the various coronary syndromes. Circulation.

[B3] Koerselman J, van der Graaf Y, de Jaegere PP, Grobbee DE (2003). Coronary collaterals: an important and underexposed aspect of coronary artery disease. Circulation.

[B4] Cooke JP (2003). NO and angiogenesis. Atheroscler Suppl.

[B5] Murohara T, Asahara T, Silver M, Bauters C, Masuda H, Kalka C, Kearney M, Chen D, Symes JF, Fishman MC, Huang PL, Isner JM (1998). Nitric oxide synthase modulates angiogenesis in response to tissue ischemia. J Clin Invest.

[B6] Zhao X, Lu X, Feng Q (2002). Deficiency in endothelial nitric oxide synthase impairs myocardial angiogenesis. Am J Physiol Heart Circ Physiol.

[B7] Smith RS, Lin KF, Agata J, Chao L, Chao J (2002). Human endothelial nitric oxide synthase gene delivery promotes angiogenesis in a rat model of hindlimb ischemia. Arterioscler Thromb Vasc Biol.

[B8] Amano K, Matsubara H, Iba O, Okigaki M, Fujiyama S, Imada T, Kojima H, Nozawa Y, Kawashima S, Yokoyama M, Iwasaka T (2003). Enhancement of ischemia-induced angiogenesis by eNOS overexpression. Hypertension.

[B9] Namba T, Koike H, Murakami K, Aoki M, Makino H, Hashiya N, Ogihara T, Kaneda Y, Kohno M, Morishita R (2003). Angiogenesis induced by endothelial nitric oxide synthase gene through vascular endothelial growth factor expression in a rat hindlimb ischemia model. Circulation.

[B10] Tesauro M, Thompson WC, Rogliani P, Qi L, Chaudhary PP, Moss J (2000). Intracellular processing of endothelial nitric oxide synthase isoforms associated with differences in severity of cardiopulmonary diseases: cleavage of proteins with aspartate vs. glutamate at position 298. Proc Natl Acad Sci USA.

[B11] Philip I, Plantefeve G, Vuillaumier-Barrot S, Vicaut E, LeMarie C, Henrion D, Poirier O, Levy BI, Desmonts JM, Durand G, Benessiano J (1999). G894T polymorphism in the endothelial nitric oxide synthase gene is associated with an enhanced vascular responsiveness to phenylephrine. Circulation.

[B12] Gibson CM, Ryan K, Sparano A, Moynihan JL, Rizzo M, Kelley M, Marble SJ, Laham R, Simons M, McClusky TR, Dodge JT (1999). Angiographic methods to assess human coronary angiogenesis. Am Heart J.

[B13] Miller SA, Dykes DD, Polesky HF (1988). A simple salting out procedure for extracting DNA from human nucleated cells. Nucleic Acids Res.

[B14] McNamara DM, Holubkov R, Postava L, Ramani R, Janosko K, Mathier M, MacGowan GA, Murali S, Feldman AM, London B (2003). Effect of the Asp298 variant of endothelial nitric oxide synthase on survival for patients with congestive heart failure. Circulation.

[B15] Casas JP, Bautista LE, Humphries SE, Hingorani AD (2004). Endothelial nitric oxide synthase genotype and ischemic heart disease: meta-analysis of 26 studies involving 23028 subjects. Circulation.

[B16] Miyamoto Y, Saito Y, Kajiyama N, Yoshimura M, Shimasaki Y, Nakayama M, Kamitani S, Harada M, Ishikawa M, Kuwahara K, Ogawa E, Hamanaka I, Takahashi N, Kaneshige T, Teraoka H, Akamizu T, Azuma N, Yoshimasa Y, Yoshimasa T, Itoh H, Masuda I, Yasue H, Nakao K (1998). Endothelial nitric oxide synthase gene is positively associated with essential hypertension. Hypertension.

[B17] Khaja F, Sabbah HG, Brymer JF, Stein PD (1988). Influence of coronary collaterals on left ventricular function in patients undergoing coronary angioplasty. Am Heart J.

[B18] Ziche M, Morbidelli L, Choudhuri R, Zhang HT, Donnini S, Granger HJ, Bicknell R (1997). Nitric oxide synthase lies downstream from vascular endothelial growth factor-induced but not basic fibroblast growth factor-induced angiogenesis. J Clin Invest.

[B19] Marsden PA, Heng HH, Scherer SW, Stewart RJ, Hall AV, Shi XM, Tsui LC, Schappert KT (1993). Structure and chromosomal localization of the human constitutive endothelial nitric oxide synthase gene. J Biol Chem.

[B20] Hingorani AD, Liang CF, Fatibene J, Lyon A, Monteith S, Parsons A, Haydock S, Hopper RV, Stephens NG, O'Shaughnessy KM, Brown MJ (1999). A common variant of the endothelial nitric oxide synthase (Glu298--> Asp) is a major risk factor for coronary artery disease in the UK. Circulation.

[B21] Fairchild TA, Fulton D, Fontana JT, Gratton JP, McCabe TJ, Sessa WC (2001). Acidic hydrolysis as a mechanism for the cleavage of the Glu(298)--> Asp variant of human endothelial nitric-oxide synthase. J Biol Chem.

[B22] Golser R, Gorren AC, Mayer B, Schmidt K (2003). Functional characterization of Glu298Asp mutant human endothelial nitric oxide synthase purified from a yeast expression system. Nitric Oxide.

[B23] McDonald DM, Alp NJ, Channon KM (2004). Functional comparison of the endothelial nitric oxide synthase Glu298Asp polymorphic variants in human endothelial cells. Pharmacogenetics.

[B24] Tanus-Santos JE, Desai M, Flockhart DA (2001). Effects of ethnicity on the distribution of clinically relevant endothelial nitric oxide variants. Pharmacogenetics.

[B25] Marroni AS, Metzger IF, Souza-Costa DC, Nagassaki S, Sandrim VC, Correa RX, Rios-Santos F, Tanus-santos JE (2005). Consistent interethnic differences in the distribution of clinically relevant endothelial nitric oxide synthase genetic polymorphisms. Nitric Oxide.

[B26] Pohl T, Seiler C, Billinger M, Herren E, Wustmann K, Mehta H, Windecker S, Eberli FR, Meier B (2001). Frequency distribution of collateral flow and factors influencing collateral channel development. Functional collateral channel measurement in 450 patients with coronary artery disease. J Am Coll Cardiol.

[B27] Werner GS, Bahrmann P, Mutschke O, Emig U, Betge S, Ferrari M, Figulla HR (2003). Determinants of target vessel failure in chronic total coronary occlusions after stent implantation. The influence of collateral function and coronary hemodynamics. J Am Coll Cardiol.

[B28] Rentrop KP, Cohen M, Blanke H, Phillips RA (1985). Changes in collateral channel filling immediately after controlled coronary artery occlusion by an angioplasty balloon in human subjects. J Am Coll Cardiol.

